# Therapeutic Effect of Alpha Linolenic Acid on Cutaneous Wound Healing in Hyperglycemic Mice: Involvement of Neurotrophins

**DOI:** 10.3390/pharmaceutics17111427

**Published:** 2025-11-04

**Authors:** Thais Paulino do Prado, Flávia Cristina Zanchetta, Aline Cristina Rosa Maria, Thaiane da Silva Rios, Guilherme Rossi de Assis-Mendonça, Maria Helena Melo Lima, Dennys Esper Correa Cintra, Joseane Morari, Lício A. Velloso, Eliana P. Araújo

**Affiliations:** 1 School of Nursing, University of Campinas, Campinas 13083-887, Brazil; thaispdoprado@gmail.com (T.P.d.P.); flaviaz@unicamp.br (F.C.Z.); melolima@unicamp.br (M.H.M.L.); 2 Obesity and Comorbidities Research Center, University of Campinas, Campinas 13083-887, Brazil; dennys@unicamp.br (D.E.C.C.); morarij@gmail.com (J.M.); lavellos@unicamp.br (L.A.V.); 3 School of Pharmaceutical Sciences, University of Campinas, Campinas 13083-887, Brazil; thaianerios2@gmail.com; 4 School of Applied Sciences, University of Campinas, Limeira 13484-350, Brazil; t148039@dac.unicamp.br; 5 School of Medical Sciences, University of Campinas, Campinas 13083-887, Brazil; grassis@unicamp.br

**Keywords:** skin, fatty acids, omega-3, osmotic pump, diabetes mellitus, BDNF, TGF-beta

## Abstract

**Background:** Alpha-linolenic acid (ALA) is an essential fatty acid from the omega-3 family that plays an important role in skin homeostasis. It is known for its anti-inflammatory properties, which can contribute to wound healing. Neurotrophins, such as Brain-Derived Neurotrophic Factor (BDNF), may also play an important role in the skin, influencing nerve regeneration and pain modulation. **Objectives:** This article aims to explore the therapeutic effect of ALA on wound healing in streptozotocin-induced hyperglycemic mice, with an emphasis on the involvement of neurotrophins. **Methods:** We used keratinocyte cultures exposed or not to ALA and male C57BL6-J mice, which were randomly divided into four groups: non-hyperglycemic treated with vehicle; non-hyperglycemic treated with ALA; hyperglycemic treated with vehicle; and hyperglycemic treated with ALA. The treatment was administered continuously via a subcutaneous osmotic pump. **Results:** We found that controlled ALA administration potentiates the wound healing process in hyperglycemic mice by accelerating the inflammatory phase and promoting early granulation tissue formation (73.2% ± 0.7 vs. 92.2% ± 2.8 on day 7, n = 5; *p* < 0.05). This is supported by the balance between the expression of vimentin, CD31, and MMP-9. Furthermore, ALA modulates proteins linked to peripheral neurogenesis and gliogenesis, such as BDNF, NTRK2, SOX-10, CNTF, CTNFR, and STAT-3. It may also promote wound healing and nerve regeneration at the wound site in hyperglycemic animals. In non-hyperglycemic mice, ALA improves the quality of scars but does not accelerate the wound healing process, even with the positive modulation of certain genes relevant to skin healing. **Conclusions:** Alpha-linolenic acid improves skin wound healing and increases gene expression related to nerve regeneration in wounds of hyperglycemic mice.

## 1. Introduction

Wound healing is a complex process that involves the regeneration of tissues following an injury. This process can be influenced by various factors, including the presence of chronic diseases such as diabetes [1,2]. In healthy subjects, the healing process follows a physiological path as the body can respond adequately to injuries, promoting the formation of a fibrin plug immediately after the loss of tissue continuity, followed by cell migration and collagen synthesis, which are essential for wound regeneration [1,2]. Factors such as proper nutrition, hydration, and effective wound care can optimize this process [3]. In contrast, patients with diabetes often encounter healing challenges. Chronic hyperglycemia can adversely affect blood vessel function and immune response, leading to slower healing and an increased risk of infections [1,2]. Studies indicate that diabetes can result in neuropathy, ischemia, and alterations in collagen synthesis, all of which hinder the healing process [4]. It is currently estimated that approximately 463 million people live with diabetes worldwide, and many of these individuals face complications related to healing [5]. In addition, a large number of patients develop Diabetic Foot Ulcers (DFUs). DFUs are chronic skin lesions characterized by hypoxia, excess reactive oxygen species (ROS), inflammatory dysregulation, and increased bacterial growth, often resistant to standard treatments. ROS play an important role in the body’s defense and wound healing; however, adequate control of their production is vital, as excess ROS can lead to dysregulation and complications in the healing process. Therefore, it is crucial to address the underlying factors that contribute to the imbalance of ROS in wounds, particularly through effective blood sugar management and dressings that could help reversing this condition [6,7].

Alpha-linolenic acid (ALA), an omega-3 fatty acid found in sources such as flaxseeds and canola oil, has shown beneficial effects on healing [8]. ALA can act reducing inflammation and improving the immune response, which is particularly important in patients with diabetes, as chronic inflammation can hinder healing [8]. In people with diabetes, its anti-inflammatory action may help mitigating some of the adverse effects of diabetes on the healing process [9,10]; nevertheless, there is still a gap of information regarding the mechanisms mediating the positive effects of ALA in this context.

Although neurotrophins, such as Brain-Derived Neurotrophic Factor (BDNF), are best known for their roles in the central nervous system, they can also promote the survival, development, and function of neurons outside the brain [11,12]. Studies have shown that they may also play an important role in the skin [13], influencing nerve regeneration, response to injury, and pain modulation [14]. In addition, neurotrophins can help regulate the inflammatory response and communicate signals between nerve cells and skin cells, contributing to the maintenance of skin health [13]. This is especially important in wound healing processes, where the interface between different cell types is necessary for effective healing.

In this study, we hypothesized that in hyperglycemic conditions the production and function of neurotrophins may be impaired, affecting healing. Treatment with ALA may improve peripheral nerve function at the wound site and healing in C57BL/6J mice. Treatment with alpha-linolenic acid (ALA) could enhance peripheral nerve function at the wound site and promote healing in C57BL/6J mice. Understanding how hyperglycemia impacts inflammation and immune function can help clarify the complications associated with wound healing in hyperglycemic conditions. C57BL/6J mice possess a defined genetic background, which minimizes genetic variability in experiments and allows for a clearer assessment of the effects on inflammation. Understanding these mechanisms can not only enrich scientific knowledge about wound healing but also pave the way for innovative and effective therapeutic approaches.

## 2. Materials and Methods

### 2.1. Cell Culture MTT

Experiments were performed using human keratinocytes (HaCaT) and human fibroblasts (BJ-5ta), purchased from the Rio de Janeiro Cell Bank, RJ, B (BCRJ/ https://bcrj.org.br). The cells were incubated at 37 °C in a 5% CO_2_ atmosphere for expansion in specific culture media [HaCaT: Dulbecco’s Modified Eagle’s Medium (DMEM) (Thermo Fisher Scientific, Waltham, MA, USA) with 4 mM L-glutamine, 4.5 g/L glucose, and 1.5 g/L sodium bicarbonate, supplemented with 10% Fetal Bovine Serum (FBS); BJ-5ta: 4 parts of DMEM with 4 mM L-glutamine, 4.5 g/L glucose, and 1.5 g/L sodium bicarbonate (Thermo Fisher Scientific, Waltham, MA,USA), combined with 1 part of Medium 199, supplemented with 0.01 mg/mL hygromycin B and 10% FBS(Sigma-Aldrich (Merck KGaA) St. Louis State, MO, USA]. After reaching 80–90% confluence, the cells were detached using a 0.25% trypsin solution and seeded into 12- or 24-well culture plates, according to the experimental groups in replicates. To verify cell viability by quantifying the number of living cells, the cytotoxicity of treatments was estimated, and cell proliferation was quantitatively detected by measuring the cell growth rate due to a linear relationship between cell activity and absorbance. The MTT (3-(4,5-dimethylthiazol-2-yl)-2,5-diphenyltetrazolium bromide) assay was performed(Thermo Fisher Scientific, Waltham, MA, USA) [15]. The cells were cultured in 12-well plates at a concentration of 5 × 10^5^ cells/well until reaching 90–100% confluence and maintained in an incubator in an atmosphere of 5% CO_2_ at 37 °C for 24 h. To perform the assay, the culture medium was removed after washing with phosphate-buffered saline (PBS 0.1 M). α-Linolenic fatty acid (Cargill, Inc.,Wayzata, MN, USA)was added to the wells at concentrations of 5 µM, 50 µM, or 100 µM in culture medium and incubated for 24, 48, or 72 h. Absolute ethanol (ETOH) P.A. (dilution solution for ALA) was used as the control at the highest concentration. Afterward, the treatment medium was removed, the wells were washed with phosphate-buffered saline (PBS 0.1 M), and the cells were incubated for two hours with MTT solution (5 mg/mL). They were then subjected to a 15-min reaction with dimethyl sulfoxide (DMSO) under light protection and gentle agitation. The absorbance was read with a spectrophotometer (brand and model) at wavelengths of 550 and 620 nm using a microplate reader (Globomax, Promega Corporation, Maadison, WI, USA).

### 2.2. Experimental Animals

The study was conducted following ARRIVE guidelines for reporting animal research. Eight-week-old male C57BL/6J isogenic mice, in the juvenile stage of maturation and weighing approximately 25 g, were used in the study. They were obtained from the vivarium of the State University of Campinas and were in perfect health and immunological condition. No specific exclusion criteria were established a priori; however, any mice showing signs of illness or distress during the study were excluded from the study. For each experimental protocol, six mice were assigned to each group, resulting in a total of 138 mice. The number of mice per experimental group was calculated using the formula n = 1 + [2C × (s/d)^2^], where C = (za + zb)^2^, s = maximum deviation, and d = expected difference between the groups. Assuming a significance level of 95%, we find C = (1.96 + 1.96)^2^ = 15.37, with s = 0.2 and d = 0.5. Thus, n = 1 + [2 × 15.37 × (0.2/0.5)^2^] = 5.97, which rounds up to n = 6. The mice were housed in individual cages enriched with tunnels and hiding places and had access to standard rodent chow (Purina Animal Nutrition, Gray Summit, MO, USA and filtered water “ad libitum,” under controlled lighting conditions (12-h light/dark cycle) and a temperature of 20 ± 2 °C. The mice were randomly divided into four groups: vehicle-treated control, ALA-treated control, vehicle-treated hyperglycemic, and ALA-treated hyperglycemic. To minimize potential confounders, a randomized block design was used, ensuring random treatment assignment within blocks of similar characteristics. The order of treatments was altered systematically, and animal and cage locations were also randomized to reduce bias and location-related effects. At the allocation stage, the researchers assigning the mice were aware of the group allocations. Personnel conducting the experiment and outcome assessors were blinded to the group assignments to prevent bias. Hyperglycemia was induced in the 8-week-old C57BL/6 mice through intraperitoneal injections of streptozotocin (STZ) (Sigma-Aldrich (Merck KGaA) St. Louis State, MI, USA) at a dose of 50 mg/kg, diluted in sodium citrate buffer at pH 4.5, for five consecutive days. After four weeks, blood samples were collected from the tail vein, and blood glucose levels were measured using a glucometer (Accu-Check, Roche Diagnostics, Indianapolis, IN, USA). The criterion for diagnosing hyperglycemia was established as a blood glucose level of ≥250 mg/dL. Furthermore, fasting blood glucose levels were measured every two days throughout the experiments. We found that hyperglycemic mice treated with ALA tended to have lower fasting blood glucose levels compared to hyperglycemic mice treated with vehicles. However, there were no significant variations observed during the experiments (Figure S1). The outcomes assessed were, as follows: wound closure and morphology; molecular markers; and the health and well-being of the animals. After the experimental period, mice were submitted to euthanasia with ketamine (300 mg/kg), xylazine (30 mg/kg), and sodium thiopental (100 mg/kg) (-). During the experimental period, the mice were monitored daily to assess food intake, fur appearance, pain, and discomfort, which were regarded as early signs of pain, suffering, or stress. Any mice exhibiting such behaviors were removed from the study based on the humane endpoint criteria.

### 2.3. Osmotic Pump

The ALZET^®^ 2006 and 2004 osmotic pumps Durect Corporation, Cupertino, CA, USA)were utilized for the continuous administration of ALA treatment until euthanasia. The pumps were handled aseptically in a laminar flow hood 48 h prior to installation, immersed in a 0.9% saline solution, and maintained at 37 °C in a 5% CO_2_ atmosphere. Following this, ALA at a concentration of 5 mM or ETOH P.A.(BASF Corporation, Florham Park, NJ, USA) was introduced using a filling tube, a specialized needle, and a 100 IU insulin syringe. The mice were anesthetized intraperitoneally, and after confirming complete loss of sensory reflexes, an incision was made in the interscapular region to insert the pump. One week later, the wounds were created. We determined the ALA concentration based on findings from an article that reported the supplementation of culture medium with 10 μM of ALA in a tissue-engineered skin model [16]. Consequently, we opted to culture with half the concentration from that study (5 μM), as well as five times that concentration (50 μM) and ten times that concentration (100 μM). The optimal result was then selected for the remainder of the study.

### 2.4. Wound

Two excisional wounds were created on the dorsal region of the mice; the skin and subcutaneous tissue were removed using a 6 mm metal biopsy punch until the muscle fascia was exposed [17]. After surgery, the mice received analgesia with tramadol hydrochloride (50 mg/kg) intraperitoneally [17].

### 2.5. Eosin and Hematoxylin Staining

The tissues were fixed and embedded in paraffin. Histological sections with a thickness of 5 µm were prepared and subsequently underwent a deparaffinization and hydration process. This involved a sequential series of phenolic solutions (xylene and ethanol) in decreasing concentrations. For staining, the sections were immersed in a hematoxylin solution after being washed with eosin dye. They then underwent a dehydration process to set the stain.

### 2.6. Picrosirius Red

Staining was conducted by immersing the samples for 60 min in a commercial dye, followed by differentiation in ethanol and mounting with synthetic resin. The samples were then evaluated using polarized light microscopy, which enables the distinction of type I collagen (red-yellow), type III collagen (green), and intermediate collagen (yellow) [18].

### 2.7. Healing Process Evaluation

Wound healing was assessed through photographs taken with a Samsung SM-G780F camera (Samsung Electronics, Suwon, GG, SK )(F1.8, 1/120 s, 5.40 mm, ISO250) on days 0, 3, 7, 12, and 21 after the injury. The images were digitized, and the wound area was measured using ImageJ^®^ software, version 1.53t. (National Institutes of Health, Bethesda, MD, USA). Wound closure was expressed as a percentage (%), calculated using the following mathematical formula: (Initial wound area) − (Daily wound area) x 100 / (Initial area).

### 2.8. RT-PCR

The samples were collected in Trizol organic solvent. Then, the genetic material was separated by the addition of chloroform and precipitation with isopropyl alcohol. The pellet of genetic material was washed until completely dehydrated with 70% ethyl alcohol and 100% ethyl alcohol. The samples were then reconstituted in Milli-Q H_2_O, and the concentration of RNA at 230 nm was quantified using Gene5 Software. A mix of cDNA synthesis containing dNTP, buffer, random primer, and sufficient water for dilution was added to ensure that the samples had the same concentration. The quantitative real-time PCR was performed using the Applied Biosystems system (Thermo Fisher Scientific (formerly Applied Biosystems), Foster City, CA, USA) with TaqMan probes.

### 2.9. Western Blot

The cells were lysed using RIPA buffer containing protease inhibitors, dNTP, and Laemmli buffer. Then, 20 µg of protein were separated by polyacrylamide gel electrophoresis (8–12%) and transferred to a nitrocellulose membrane. The membranes were blocked and incubated with specific primary antibodies, followed by an appropriate secondary antibody linked to peroxidase. A chemiluminescence kit was used to visualize the immunoreactive bands. For the Western blot analyses, the internal control was conducted using a rabbit polyclonal anti-beta-actin antibody (dilution 1:5000; Sigma-Aldrich,(Merck KGaA (Sigma-Aldrich brand), Darmstadt, HE, Germany).

### 2.10. Mass Spectrometry

Skin samples were subjected to sorptive tape-like extraction laser desorption ionization coupled with direct-print imaging mass spectrometry on a silica gel plate (60 Å) for thin-layer chromatography (Merck KGaA, Darmstadt, HE, Germany), as previously described [19]. Metabolic fingerprinting of free fatty acids was performed using a MALDI-LTQ-XL instrument with tissue imaging capability (Thermo Fisher, San Jose, CA, USA). Data acquisition for the survey scan was performed in the m/z range of 150 to 600 in negative ion mode. No matrix was applied.

### 2.11. Protein–Protein Interactions

The study of interactions was conducted on the STRING platform for protein–protein interaction analysis (https://string-db.org/). The desired organism was selected, and the names or IDs of the proteins of interest were entered in the search bar. A search was performed to visualize the interaction network, where proteins are represented as nodes and interactions as lines. Filtering options were used to adjust the visualization and nodes to obtain detailed information.

### 2.12. Statistical Analysis

Levine’s test was applied to verify the homogeneity of variances. For independent samples, Student’s *t*-test was used, and for more than two variables, analysis of variance (ANOVA) was performed. When necessary, Tukey’s test for multiple comparisons was used. Summary statistics, including means and standard deviations (or medians and ranges where applicable), were calculated for each experimental group to provide an overview of the data. Additionally, effect sizes with confidence intervals were computed to quantify the magnitude of differences between groups. In all cases, the significance level for rejection of the null hypothesis was set at 5% (*p* < 0.05). Data were analyzed using GraphPad Prism, version 8.0.1 (Software, San Diego, CA, USA)

## 3. Results

### 3.1. 5 µM of α-Linolenic Acid (ALA) Leads to Increased Keratinocyte Viability

**Figure 1:**
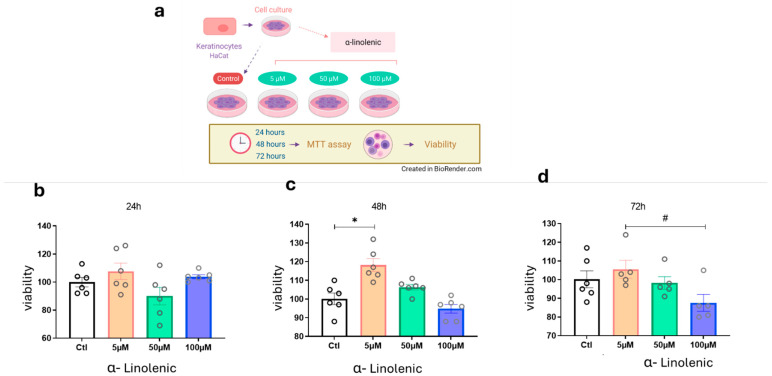
Effects of ALA Treatment on Cell Viability in Keratinocytes: (**a)** Experimental design of HaCaT cultures treated with varying concentrations of ALA for MTT viability testing; (**b**) Cell viability after 24 h of ALA exposure; (**c**) Cell viability after 48 h of ALA exposure; (**d**) Cell after 72 h of ALA exposure. Data are presented as mean ± SEM; n = 6; * *p* < 0.01; # *p* < 0.05 in one-way ANOVA. Created in BioRender. Pereira de Araujo, E. (2025) https://BioRender.com/0tmejpe.

First, we tested the hypothesis that ALA could stimulate the proliferation and survival of HaCaT cells even under confluent culture conditions (Figure 1a). All traces of fetal bovine serum were removed from the medium to attenuate the effects of growth factors present in the serum. After 24 h of exposure to ALA, there was no modulation of the cell survival pattern (Figure 1b). However, after 48 h of exposure to ALA at a concentration of 5 µM and under 100% confluence conditions, keratinocyte viability and proliferation increased (Figure 1c). This was not observed at concentrations of 50 µM and 100 µM. Since the 50 µM and 100 µM ALA concentrations showed opposite effects in the viability test (proliferative and excitotoxic, respectively), we evaluated whether keratinocyte proliferation could be affected after 72 h of exposure to ALA (Figure 1d). We then confirmed that at a concentration of 5 µM, the cells remained viable, but there were no significant differences between the groups (Figure 1).

### 3.2. α-Linolenic Acid (ALA) Leads to Increased Protein Expression of BDNF and Gene Expression of This Receptor in Keratinocytes

**Figure 2:**
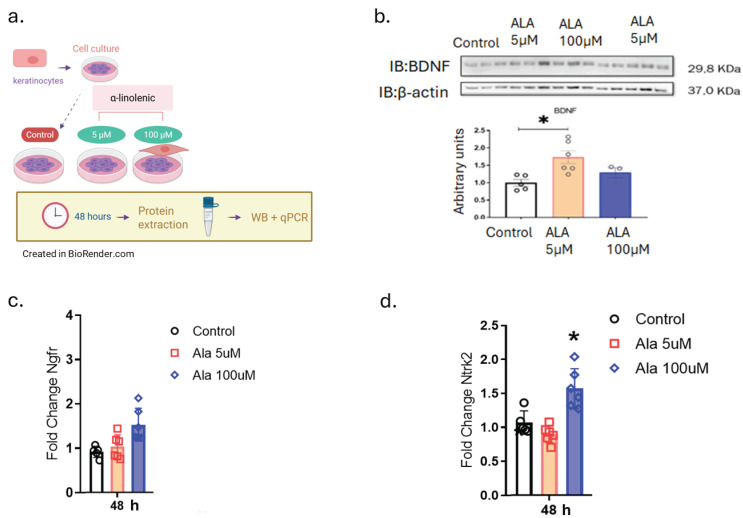
Effects of ALA on the Modulation of Brain-Derived Neurotrophic Factor (BDNF) and Its Receptors: (**a**) Experimental design of HaCaT cells treated with 5 and 100 µM ALA for 48 h; (**b**) Immunoblot determination of BDNF expression on HaCaT cells treated with 5 and 100 µM ALA for 48 h; (**c**) Real-time PCR transcript determination of Ngfr expression on HaCaT cells treated with 5 and 100 µM ALA for 48 h; (**d**) Real-time PCR transcript determination of Ntrk2 expression on HaCaT cells treated with 5 and 100 µM ALA for 48 h. Data are presented as mean ± SEM; n = 6; * *p* < 0.05 versus control in one-way ANOVA. Created in BioRender. Pereira de Araujo, E. (2025) https://BioRender.com/0tmejpe.

The next step was to evaluate the effect of ALA on the modulation of neurotrophins and their receptors (Figure 2). The choice for keratinocytes was based on a prior study that described the expression of neurotrophins by this type of cell [12]. The results show that at a concentration of 5 µM ALA there is positive modulation of BDNF protein expression and at 100 µM there is an increase in the gene expression of tropomyosin receptor kinase B (Ntrk2), but not of Ngfr in keratinocytes (Figure 2b–d).

### 3.3. α-Linolenic Acid (ALA) Leads to an Increase in the Gene Expression of Cytokines and Growth Factors in Keratinocytes

**Figure 3:**
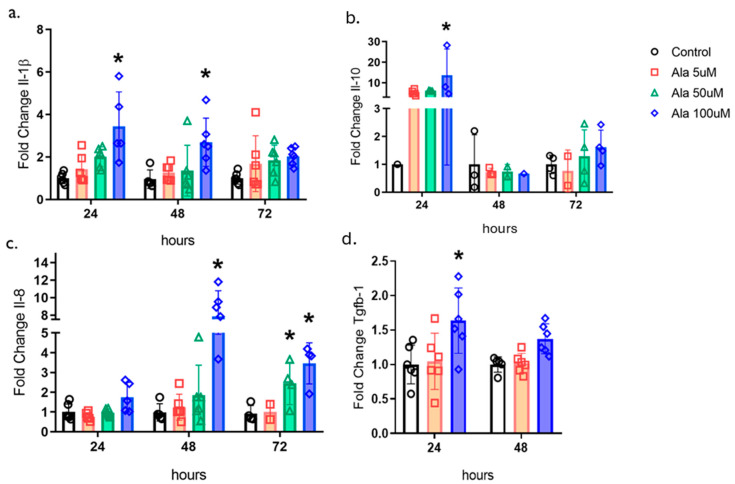
Effects of ALA on the Modulation of Cytokines and Transforming Growth Factor-β1 in Keratinocytes: (**a**) Real-time PCR transcript determination of Il1b expression on HaCaT cells treated with 5, 50 or 100 µM ALA for 24, 48, or 72 h; (**b**) Real-time PCR transcript determination of Il10 expression on HaCaT cells treated with 5, 50 or 100 µM ALA for 24, 48, or 72 h; (**c**) Real-time PCR transcript determination of Il8 expression on HaCaT cells treated with 5, 50 or 100 µM ALA for 24, 48, or 72 h; (**d**) Real-time PCR transcript determination of Tgfb1 expression on HaCaT cells treated with 5 or 100 µM ALA for 24 or 48 h. Data are presented as mean ± SEM; n = 6; * *p* < 0.05 versus control in one-way ANOVA.

To understand the effects of ALA on the molecular modulation of the healing process, we examined the gene expression of cytokines and growth factors in human keratinocytes (Figure 3). We found that ALA resulted in an increase in the gene expression of pro-inflammatory cytokines such as Il-1β and Il-8, as well as an increase in the anti-inflammatory cytokine IL-10, in addition to an increase in Tgf-β1 at a concentration of 100 µM (Figure 3a–d).

### 3.4. α-Linolenic Acid (ALA) Accelerates Skin Wound Healing in Hyperglycemic Mice

**Figure 4:**
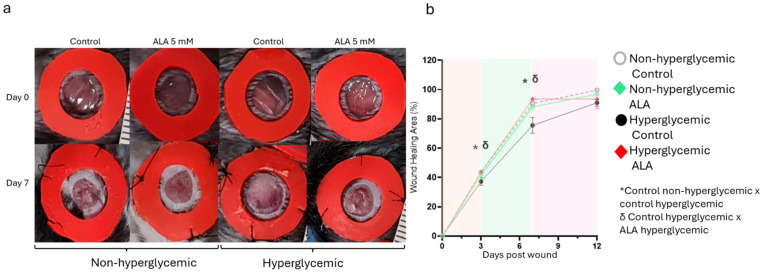
Effects of ALA on Wound Healing Modulation in Non-Hyperglycemic and Hyperglycemic Mice: (**a**) Macroscopic evaluation of wounds on days 0 and 7, comparing treatment with 5 mM ALA versus control (vehicle) in both hyperglycemic and non-hyperglycemic mice; (**b**) Imaging measurements illustrating the progression of healing from day 0 to day 12. The images are representative of five independent experiments, with a total sample size of n = 5 per group. Statistical significance was determined at * δ *p* < 0.05 using one-way ANOVA, comparing ALA treatment to the vehicle, highlighting the efficacy of ALA in promoting wound healing in both conditions.

The effect of ALA delivery on wound healing was tested during a 12-day protocol (Figure 4). As illustrated in Figure 4a,b, wound healing in hyperglycemic mice was significantly reduced compared to non-hyperglycemic mice. However, controlled delivery of ALA resulted in accelerated wound healing in hyperglycemic mice, leading to significant re-epithelialization on days 3 and 7 (32% ± 5 vs. 48% ±7 on day 3 and 73.2% ± 0.7 vs. 92.2% ± 2.8 on day 7, n = 5; *p* < 0.05). Nonetheless, this effect was not observed in non-hyperglycemic mice compared to the control group.

### 3.5. α-Linolenic Acid (ALA) Is Responsible for Modulating the Gene Expressions Involved in Wound Healing

**Figure 5:**
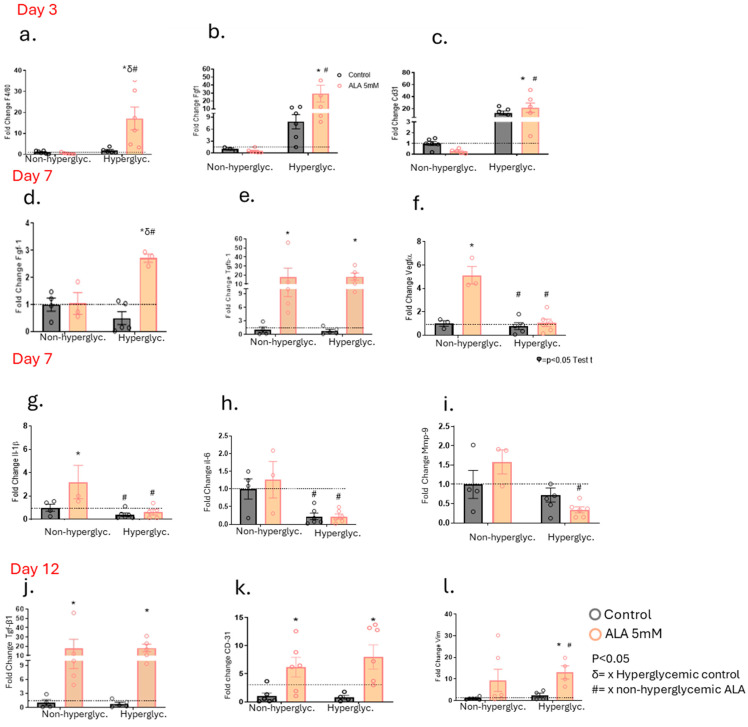
Effects of ALA on the Modulation of Gene Expression Involved in Wound Healing in Hyperglycemic and Non-Hyperglycemic Mice Treated with ALA: (**a**–**c**) Transcript expression of F4/80 (**a**), Fgf1 (**b**) and Cd31 (**c**) on day 3 post-injury; (**d**–**f**) Transcript expression of Fgf1 (**d**), Tgfb1 (**e**) and Vegfa (**f**) on day 7 post-injury; (**g**–**i**) Transcript expression of Il1b (**g**), Il6 (h) and Mmp9 (**i**) on day 7 post-injury; (**j**–**l**) Transcript expression of Tgfb1 (**j**), Cd31 (**k**) and Vim (**l**) on day 12 post-injury. Data are presented as mean ± SEM, with a sample size of N = 5–6 per group. Statistical significance was assessed using one-way ANOVA, comparing control versus ALA at 5 mM, with *p* < 0.05. δ vs. hyperglycemic control. # vs. non-hyperglycemic ALA. * vs. other groups.

After identifying the synergistic modulatory effects of ALA on other fatty acids with important functions in wound healing, we investigated the modulation of gene expression of glycoproteins, enzymes, cytokines, chemokines, and growth factors in the wounds of non-hyperglycemic and hyperglycemic animals. On day 3 post-wounding, we observed an increase in F4/80 gene expression in mice treated with ALA compared to their controls in the hyperglycemic group (Figure 5a). Additionally, there was an increase in Fgf-1 gene expression in the hyperglycemic group, regardless of ALA treatment (Figure 5b). CD-31, a marker for endothelial cells, was elevated in hyperglycemic mice, whether treated with ALA or not (Figure 5c). Furthermore, on day 7 post-wounding, Fgf-1 gene expression increased in hyperglycemic mice treated with ALA; this increase was not observed in hyperglycemic controls or in the non-hyperglycemic group (Figure 5d). Tgf-β1 gene expression was elevated in both non-hyperglycemic and hyperglycemic mice treated with ALA compared to their respective controls (Figure 5e). Similarly, Vegf expression increased only in the non-hyperglycemic group treated with ALA, relative to its control and the hyperglycemic mice (Figure 5f). Il-1β levels were higher in the non-hyperglycemic control group and in the ALA-treated group compared to the hyperglycemic group, regardless of whether they were treated with ALA or not (Figure 5g). Likewise, IL-6 levels decreased in both hyperglycemic groups—treated or not with ALA—when compared to the non-hyperglycemic group (Figure 5h). Similarly, Mmp-9 expression was reduced in hyperglycemic mice treated with ALA (Figure 5i). On day 12 post-wounding, Tgf-β1 gene expression was once again elevated in both non-hyperglycemic and hyperglycemic animals treated with ALA compared to their respective controls (Figure 5j). Additionally, CD-31 expression was increased in both groups of animals treated with ALA (Figure 5k). Vimentin levels were elevated in animals treated with ALA (Figure 5l).

### 3.6. α-Linolenic Acid (ALA) Increases Gene Expression Involved in the Survival and Growth of Neurons in Wounds of Hyperglycemic and Non-Hyperglycemic Mice

**Figure 6:**
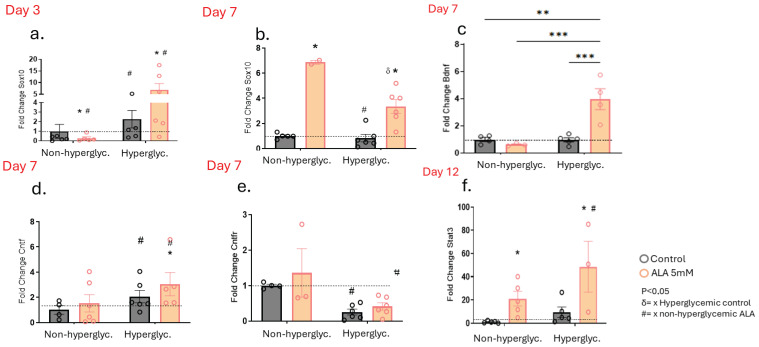
Effects of ALA on the Modulation of Genes Involved in the Survival and Growth of Neurons in Wounds of Hyperglycemic and Non-hyperglycemic Mice: (**a**) Transcript expression of Sox10 on day 3 post-injury; (**b**–**e**) Transcript expression of Sox10 (**b**), Bdnf (**c**), Cntf (**d**) and Cntfr (**e**) on day 7 post-injury; (**f**) Transcript expression of Stat3 on day 12 post-injury. Data are presented as mean ± SEM, with a sample size of n = 4–5 per group. Statistical significance was assessed using one-way ANOVA, comparing control versus ALA at 5 mM, with * *p* < 0.05. ** *p* < 0.01. *** *p* < 0.001. δ vs. hyperglycemic control. # vs. non-hyperglycemic ALA. * vs. other groups.

Next, we investigated whether, as in the cells, wounds treated with ALA would also undergo changes in the expression of genes involved in peripheral nerve regeneration. We observed an increase in Sox-10 gene expression on day 3 in the hyperglycemic group, regardless of whether they were treated with ALA or not (Figure 6a). On day 7, there was an increase in Sox-10 expression in both non-hyperglycemic and hyperglycemic groups treated with ALA (Figure 6b). Furthermore, on day 7, we observed an increase in Bdnf gene expression in the ALA-treated hyperglycemic group (Figure 6c), along with an increase in Cntf expression in both non-hyperglycemic and hyperglycemic animals treated with ALA (Figure 6d). Additionally, Cntf receptor expression was decreased in hyperglycemic mice, independent of treatment (Figure 6e). Moreover, on day 12, we observed an increase in Stat-3 gene expression in both groups treated with ALA compared to their respective controls (Figure 6f).

### 3.7. Genes Modulated in the Wounds of Hyperglycemic Mice After Treatment with α-Linolenic Acid Regulate Essential Functions in Nerve Regeneration

**Figure 7:**
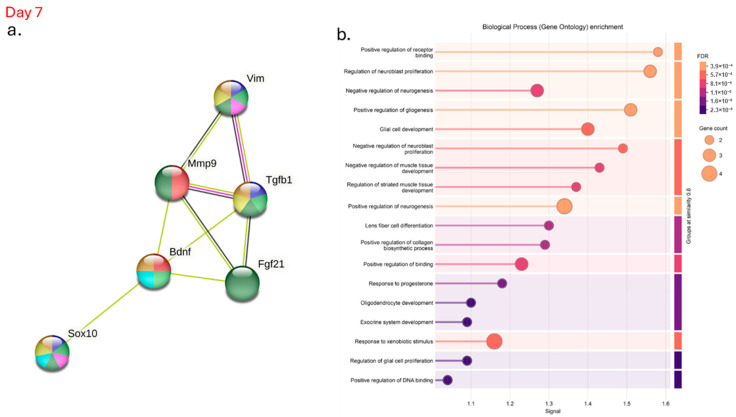
Interactome of Genes Modulated After α-Linolenic Acid (ALA) Treatment on Day 7 in Hyperglycemic Mice: (**a**) Visualization of the protein–protein interaction network demonstrating key interactions among genes modulated by ALA treatment; (**b**) Gene Ontology analysis highlighting the biological processes and molecular functions affected by ALA. Data were analyzed using the STRING database (https://string-db.org), providing insights into the complex regulatory networks underlying ALA’s effects in hyperglycemic conditions.

After evaluating the genes involved in the survival and growth of neurons, we investigated the protein–protein interaction network (Figure 7), which indicates known and predicted interactions involving increased gene expression, including those from curated databases, gene co-expression, and protein homology. There was an association of effects and co-expression between vimentin, Mmp-9, FG21, TGF-β1, SOX-10 and BDNF on day 7 in the hyperglycemic mice (Figure 7a, green and black lines). In addition, there are interactions between vimentin, TGF-β1, and MMP-9, which were identified in the interactome; the pink line indicates that the data were confirmed through experimental research (Figure 7a, pink lines). Gene Ontology enrichment highlights the biological processes associated with BDNF regulation during wound healing. The genes studied in this interactome are significantly involved in the regulation of neuroblast proliferation, as well as in the positive regulation of gliogenesis and neurogenesis on day 7 in hyperglycemic animals treated with ALA (Figure 7b).

### 3.8. α-Linolenic Acid (ALA) Improves Dermal Organization and Vascularization in Hyperglycemic Wounds at Day 7 Post-Injury

**Figure 8:**
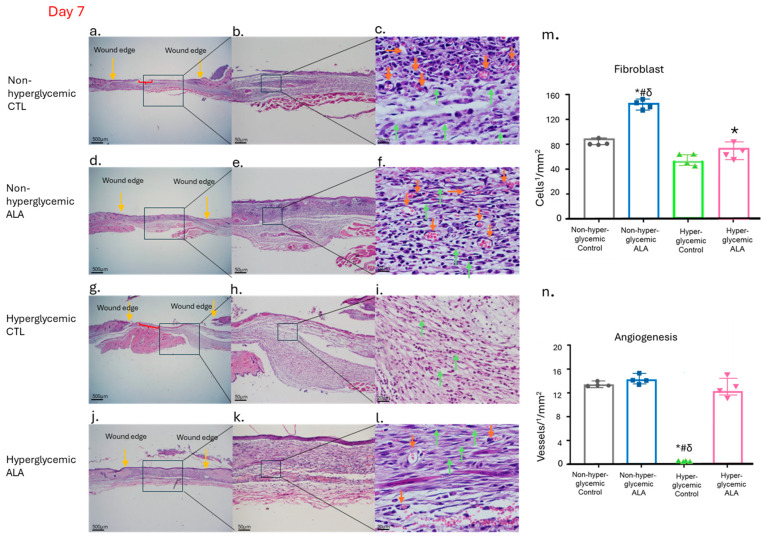
Effects of ALA on the Modulation of Newly Formed Skin Tissue 7 Days After Wounding: (**a**–**c**) Images of wounds from non-hyperglycemic animals treated with vehicle (Control), exhibiting expected tissue regeneration characterized by inflammatory infiltrate and granulation tissue formation, including visible fibroblasts and blood vessels; (**d**–**f**) wounds from non-hyperglycemic animals treated with ALA, displaying a similar pattern of tissue regeneration as observed in vehicle-treated non-hyperglycemic animals; (**g**–**i**) wounds from hyperglycemic animals treated with vehicle, demonstrating delayed healing, with wounds remaining in the inflammatory phase; (**j**–**l**) wounds from hyperglycemic animals treated with ALA, showing enhanced healing characteristics, with wound appearance nearly comparable to that of non-hyperglycemic animals; (**m**) fibroblast count; (**n**) blood vessel count per1/4 ¼ mm^2^ of tissue across different groups, showing a significant increase in fibroblasts in both non-hyperglycemic and hyperglycemic groups treated with ALA and once again, a delay in the healing process, with the maintenance of the inflammatory process. Yellow arrows indicate the wound edge, orange arrows denote blood vessels, green arrows highlight fibroblasts, red braces illustrate areas of epidermal discontinuity, and the black square marks the region represented in the subsequent figure. Magnification is 500 µm in (**a**,**d**,**g**,**j**); 50 µm in (**b**,**e**,**h**,**k**); and 20 µm in (**c**,**f**,**i**,**l**). Data are representative of n = 4 for each group. Statistical significance was assessed using one-way ANOVA, comparing hyperglycemic control versus hyperglycemic ALA at 5 mM, # non-hyperglycemic control, δ non-hyperglycemic ALA with * *p* < 0.05.

We also evaluated the characteristics of the tissue formed 7 days after the wounding using hematoxylin/eosin (Figure 8). We observed that in non-hyperglycemic mice, both in the control group and in the group treated with ALA, the newly formed tissue did not show significant changes regarding inflammatory infiltrate and the formation of new vessels (Figure 8a–f). Meanwhile, in hyperglycemic mice treated with ALA, the dermis was better structured, with a greater number of fibroblasts and less inflammatory infiltrate, as well as a greater number of vessels (Figure 8g–l). We also observed the formation of a thin layer of epidermis in both non-hyperglycemic and hyperglycemic animals treated with ALA, which did not occur in those not treated with ALA (Figure 8).

### 3.9. α-Linolenic Acid (ALA) Enhances Type I Collagen Deposition in Hyperglycemic Wounds During Critical Phases of Wound Healing

**Figure 9:**
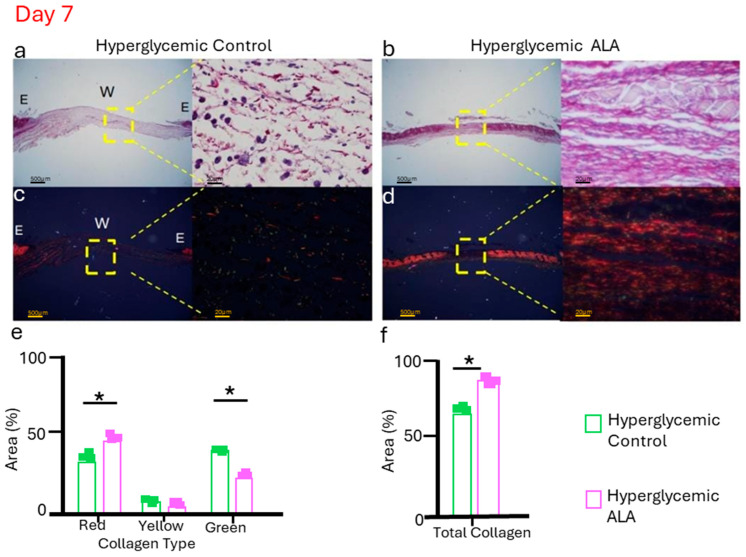
Representative picrosirius red staining was evaluated without polarized microscopy (**a**,**b**), and with polarized light (**c**,**d**), for hyperglycemic control and hyperglycemic ALA wounds on day 7, respectively. Hyperglycemic ALA wounds show collagen fibers that are more organized and thicker than those of hyperglycemic control wounds. In (**e**), it is observed that hyperglycemic ALA wounds present a significantly larger quantity of collagen type I (red) compared to the controls, while hyperglycemic control wounds show more collagen type III (green). In (**f**), it is demonstrated that hyperglycemic ALA wounds exhibit a significantly larger quantity of total collagen when compared with the controls. Data are presented as mean ± SEM (n = 3 per group). Representative histology is shown in bright fields and polarized light microscopy, with scale bars of 500 μm and 20 μm. Statistical analysis was performed using one-way ANOVA followed by Tukey’s post-hoc test, comparing hyperglycemic control versus hyperglycemic ALA at 5 mM with * *p* < 0.05.

As the treatment with ALA resulted in more efficient healing, we evaluated collagen deposition by comparing hyperglycemic control mice with ALA-treated mice on day 7, the time point at which skin recovery after injury is most challenging. In brightfield imaging, the ALA-treated group exhibited collagen fibers that appeared as thicker and more organized bundles, while in the hyperglycemic control group, the inflammatory infiltrate was more evident than collagen deposition (Figure 9a,b). Under polarized light, the hyperglycemic ALA group showed more consistent deposition of type I (mature) collagen fibers (red) compared to the control group, which had a greater number of type III (immature) collagen fibers (green) (Figure 9c,d). When quantifying collagen, we found that the hyperglycemic group treated with ALA exhibited a greater number of mature fibers (red), while the hyperglycemic control group showed a higher quantity of immature fibers (green) (Figure 9e). In terms of total collagen, the ALA-treated group demonstrated a significant increase in collagen deposition compared to the hyperglycemic group treated with vehicle, further highlighting the beneficial effects of the treatment (Figure 9f).

## 4. Discussion

Prior studies in the field have shown that α-linolenic acid (ALA) has pleiotropic effects on neuroprotection, neuroplasticity, and the maintenance of skin homeostasis [20]. Here, we hypothesized that ALA could modulate neurotrophins as well as growth factors and cytokines important for wound healing and nerve regeneration. To explore this question, we first studied cell viability after the stimulatory effect of ALA (Figure 1). We found that ALA at a concentration of 5 µM can induce the survival of human keratinocytes (Figure 1b–d), and treatment with ALA led to an increase in the protein expression of BDNF (Figure 2b). Studies have shown that one of the main effects of ALA on neuroprotection in the central nervous system may be mediated through increased BDNF levels, a protein extensively expressed in the brain that plays essential roles in neuronal preservation [21]. O’Brien et al. found that mice with diabetes-induced peripheral neuropathy exhibited reduced BDNF expression in the foot skin and, subsequently, decreased expression of the BDNF receptor TrkB, which is primarily associated with promoting survival, differentiation, and growth in neurons [12]. In our study, we observed that ALA at 5 µM leads to increased BDNF protein expression, while at a concentration of 100 µM, it enhances the gene expression of its receptor TrkB2, while it did not modulate the expression of NGFr, a BDNF receptor involved in the apoptotic pathway, in wild-type human keratinocytes (Figure 2b–d).

We then hypothesized that if we evaluated the cytokines in keratinocyte cultures stimulated with ALA, we might find similar important results that could be translated to animal studies (Figure 3). We observed that keratinocytes stimulated with ALA showed an increase in the gene expression of IL-1β, IL-10, IL-8 (CXCL8), and TGF-β1 at a concentration of 100 µM (Figure 3a–d). In this experiment, conducted with isolated cells that are not exposed to the influences of the complete “in vivo” mechanism, we observed that the modulation of cytokines, both pro-inflammatory and anti-inflammatory, occurred simultaneously. All these proteins play a vital roles in different phases. In the inflammatory phase, the chemokine CXCL8 acts as a chemotactic factor, initiating the migration of neutrophils to the wound site [22]. IL-1β has a double function; it is required in the initial inflammatory phase for the removal of debris and enrollment of growth factors. However, at the end of the process, it can exert damaging actions [23]. Conversely, IL-10 has a predominant anti-inflammatory function and prevents excessive collagen deposition and fibrosis [24]. TGF-β1 is important in re-epithelialization, inflammation, angiogenesis, and the formation of granulation tissue [25]. All these factors are fundamental for the formation of new epithelial tissue near the uninjured skin.

In the second part of the study, we evaluated the impact of ALA on wounds made on mice. Initially, we determined how much ALA and its derivatives were reaching the lesion site through lipidomics. We found no significant differences in the concentrations of total lipids, saturated fatty acids (SFA), monounsaturated fatty acids (MUFA), polyunsaturated fatty acids (PUFA), omega-6 (ω6), omega-3 (ω3), and the ratio of ω6 to ω3 (g) in tissues treated with 5 mM ALA compared to control mice, hyperglycemic mice, and hyperglycemic mice treated with ALA. These results indicate that the delivery system is safe (Figure S2).

Next, we assessed the effects of ALA on wound healing (Figure 4). We observed that ALA led to accelerated wound healing from days 3 to 7 post-injury in hyperglycemic animals treated with ALA compared to those treated with the vehicle (Figure 4a,b). Several studies have demonstrated the positive effects of linolenic acid on skin wounds. Rats treated with topical ALA exhibited more than a 60% reduction in necrotic cell layer thickness compared to controls [26]. Furthermore, a study in rodents demonstrated an increased number of neutrophils at the wound site treated with ALA compared to the control group [27]. Additionally, a study found that orally administered ALA improved wound healing in streptozotocin-induced diabetic rats [28]. A randomized clinical trial showed that participants with diabetic foot ulcers who were supplemented with ALA (1000 mg of omega-3 fatty acids from flaxseed oil supplements taken twice daily for 12 weeks) had a significantly improved wound healing compared to controls [29]. In our case, the animals reestablished the normal healing pattern, presenting re-epithelialization very similar to that of the non-hyperglycemic mice (Figure 4b). The presence of fatty acids in the blood after continuous subcutaneous administration indicates effective absorption and consistent release, resulting in stable plasma levels and more predictable absorption. The fatty acids are rapidly distributed to the tissues due to increased blood flow at the infusion site, and their metabolism primarily occurs in peripheral tissues. Topical administration may lead to minimal absorption based on wound characteristics, while oral administration may result in slower distribution due to hepatic transport and metabolism.

Subsequently, we explored the effects of ALA on the modulation of growth factors. Once again, we observed the promising effects of controlled delivery of ALA on wound healing (Figure 5). There was a significant increase in the gene expression of Fgf-1 on day 3, as well as Fgf-1 and Tgf-β1 on day 7, and Tgf-β1 on day 12 in hyperglycemic animals treated with ALA (Figure 5b,d,e,j). Whereas in non-hyperglycemic mice treated with ALA, there was only an increase in Tgf- β1 on days 7 and 12 after injury. It is well-established that these growth factors play a fundamental role in the healing process. Fgf-1 is responsible for promoting cell proliferation, migration, and differentiation, particularly in the epidermis, dermis, and blood vessels. It is crucial in the process of angiogenesis, which is essential for supplying oxygen and nutrients to the healing wound [30,31]. TGF-β exerts pleiotropic effects on wound healing by regulating cell proliferation, differentiation, extracellular matrix (ECM) production, and modulating the immune response. This phenomenon is important for inflammation, angiogenesis, granulation tissue formation, and re-epithelialization [25,32]. This suggests that ALA not only stimulates the early response to injury but also supports sustained growth factor expression, which is crucial for effective healing in hyperglycemic conditions. In contrast, in non-hyperglycemic mice treated with ALA, the increase in growth factor expression was more limited, with only Tgf-β1 showing elevated levels on days 7 and 12 post-injury. This disparity indicates that while ALA promotes growth factor expression in both groups, its effects are more pronounced in hyperglycemic animals, where the need for enhanced healing mechanisms may be greater due to underlying metabolic challenges.

Conversely, we observed a decrease in the gene expression of VEGF on day 7, along with an increase in Cd31 on days 3 and 12 in the hyperglycemic group treated with ALA (Figure 5c,f,k). All two proteins are involved in the formation of new blood vessels in the wound. The VEGF factor stimulates angiogenesis and facilitates epithelialization [33] and CD31 is a cell adhesion molecule expressed in endothelial cells that promotes angiogenesis [34]. These data suggest that despite the decrease in Vegf, Cd31 may be facilitating angiogenesis, as we did not observe any delay in this process. This phenomenon may occur through compensatory or alternative mechanisms that do not exclusively rely on VEGF. Thus, it is possible that ALA activates other signaling pathways that promote angiogenesis, involving additional growth factors or inflammatory mediators, specifically under hyperglycemic conditions, since in normoglycemic conditions there was an increase in Vegf and Cd-31 on days 7 and 12 respectively in non-hyperglycemic mice treated with ALA. Although the results appear to be contradictory between the non-hyperglycemic and hyperglycemic groups, our studies using H/E staining showed an increase in the expression of blood vessels in the granulation tissue of hyperglycemic and non-hyperglycemic mice treated with ALA (Figure 8f,l).

Furthermore, the significant increase in F4/80 expression in hyperglycemic mice treated with ALA on day 3 is an important phenomenon for healing (Figure 6a). F4/80 is a marker used to identify macrophages, which play a crucial role in the initial phase of healing, characterized by inflammation.

The gene expressions of Il-1β and Il-6 on day 7 post-injury (Figure 5g,h) were quite intriguing. In the non-hyperglycemic mice, the increase in the expression of these pro-inflammatory cytokines suggests that ALA may be enhancing the inflammatory response, potentially promoting mechanisms that depend on inflammation to initiate healing processes. This heightened inflammatory response could be beneficial in a non-hyperglycemic context, where the healing environment is typically more favorable. Conversely, the decrease in the gene expression of Il-1β and Il-6 in hyperglycemic mice after treatment with ALA indicates a possible reduction in the chronic inflammatory state often observed in hyperglycemic environments. Chronic inflammation can hinder the healing process, so the ability of ALA to downregulate these cytokines in hyperglycemic mice may contribute to an improved healing environment.

Vimentin is a cytoskeletal protein fundamental for the proliferative and remodeling phases of wound healing. It is required for TGF-β signaling, fibroblast activity, and collagen deposition [35]. In our study, vimentin gene expression was increased in hyperglycemic animals treated with ALA on day 12, which is precisely when the activity of this protein is necessary for wound remodeling (Figure 5l. Finally, we observed a decrease in Mmp-9 in hyperglycemic mice treated with ALA, which we found to be quite appropriate (Figure 5i. Although MMP-9 plays a complex role in wound healing, high levels are often associated with delayed healing, particularly in hyperglycemic wounds. MMP-9 is involved in keratinocyte migration and angiogenesis, but its excessive presence can degrade collagen and hinder adequate tissue repair [36].

To test our hypothesis that ALA, similar to its role in the central nervous system, may also be involved in the activation of nerve regeneration and growth in the peripheral nervous system, we evaluated the expression of Sox-10. We observed an increase on day 3 and day 7 in post-injury in hyperglycemic mice treated with ALA, and on day 7 in non-hyperglycemic animals treated with ALA, which is an important finding (Figure 6a,b). SOX-10 is a crucial transcription factor involved in the development and function of the peripheral nervous system, particularly in the differentiation of neural glial cells such as Schwann cells and Satellite cells. It is essential for maintaining the identity and proper development of these cells, and mutations in SOX-10 can lead to various neurological disorders, including peripheral neuropathies [37,38]. Additionally, SOX-10 is involved in wound healing processes; depletion of SOX-10 can delay wound healing and tissue regeneration [39]. Therefore, these findings suggest that in addition to rapid healing, ALA also modulates nerve regeneration in the scars treated with ALA, and the increase in Bdnf in this tissue (Figure 6c) corroborates the findings related to Sox-10. BDNF is a critical protein in the nervous system that supports neuronal survival, promotes neurogenesis, and enhances synaptic plasticity [21]. Inflammation can influence BDNF levels, and conversely, BDNF can modulate inflammatory processes. Elevated levels of pro-inflammatory cytokines can lead to a decrease in BDNF expression, which may contribute to neurodegenerative diseases [21]. Our treatment may decrease the expression of certain cytokines, which could have influenced the increase in Bdnf, specifically in hyperglycemic animals treated with ALA, which are inherently more inflamed at baseline. On the other hand, BDNF has neuroprotective properties and can help mitigate the effects of inflammation, promoting neuronal survival and reducing apoptotic pathways [21]. In our study, we observed that Bdnf levels were increased both in keratinocyte cultures and in ALA-treated animals (Figure 2b and Figure 6c). Additionally, the receptor that promotes cell survival, Ntrk2, was upregulated, while the receptor that leads to apoptosis, Ngfr, was downregulated (Figure 2b–d).

Another important neurotrophins in maintaining nerve homeostasis is CNTF. Cntf was increased only in non-hyperglycemic animals treated with ALA, while hyperglycemic animals showed only a tendency for increase (Figure 6d). However, we believe that the sample was small and that this could have been significant with a larger sample. However, Cntf receptor increased in hyperglycemic animals treated with ALA compared to their control group (Figure 6e). CNTF and its receptor are proteins expressed in high concentrations in peripheral nerves, and this signaling pathway is important for the survival and regeneration of damaged neurons [40,41,42]. These findings further suggest that ALA has positive effects through BDNF and CNTF, playing a beneficial role in both wound healing and peripheral nerve regeneration.

Signal Transducer and Activator of Transcription 3 (STAT-3) activation is essential in Schwann cells (the glial cells of the peripheral nervous system), as well as in axon myelination and nerve regeneration. Following nerve injury, Schwann cells activate STAT-3, which promotes cell survival and axon proliferation [43,44]. In our study, we observed an increase in Stat-3 in both non-hyperglycemic and hyperglycemic mice treated with ALA (Figure 6f), suggesting the involvement of this nerve repair pathway.

Therefore, ALA treatment appears to play a role in nerve regeneration, as we observed an increase in BDNF expression in vitro and in vivo exposed to ALA, along with increases in Sox10, Cntfr, and Stat-3.

Analysis of the data obtained from the interactome reveals a significant association between vimentin, MMP-9, FGF-21, TGF-β1, SOX-10, and BDNF in hyperglycemic mice treated with ALA on day 7 post-injury, as evidenced by the green and black lines in Figure 7a. This co-expression suggests that these factors may work together to influence critical biological processes during wound healing. Furthermore, the observed interactions between vimentin, TGF-β1, and MMP-9, confirmed through experimental investigation and indicated by the pink line in the same figure, reinforce the findings of the interactome. Gene ontology enrichment highlights the effects of ALA on biological processes such as nerve and skin regeneration, emphasizing the central role of these neurotrophins in neuroblast proliferation, as well as in the upregulation of gliogenesis and neurogenesis in ALA-treated hyperglycemic animals on day 7 (Figure 7b). Parfejevs et al. demonstrated that injury-activated glial cells enhance the expression of several secreted factors previously linked to wound healing and facilitate the differentiation of myofibroblasts through paracrine modulation of TGF-β signaling. These findings suggest that the modulation of these factors may be a promising strategy to improve neural repair and wound healing in hyperglycemic conditions.

Finally, to confirm that the reepithelialization of the cutaneous tissue was different between the studied groups, we performed hematoxylin and eosin staining and picrosirius red staining (Figure 8 and Figure 9). In hyperglycemic mice treated with ALA, tissue restoration was more advanced than in those treated with vehicle, exhibiting more blood vessels (orange arrows) and a greater quantity of fibroblasts (green arrows) (Figure 8g–l). Additionally, we also observed the formation of a thin layer of epidermis in both non-hyperglycemic and hyperglycemic mice treated with ALA, which did not occur in those not treated with ALA (red braces) (Figure 8a,b,j,k). In quantitative analysis, the same cellular pattern described in the semi-quantitative analysis was found (Figure 8m,n). Fibroblasts are crucial for rebuilding the extracellular matrix, and angiogenesis is essential for delivering nutrients and oxygen to the healing area [45]. Enhancing both vascularization and the number of fibroblasts in damaged tissue, possibly via injury-activated glial cells and TGF-β1 signaling, may lead to faster and more effective tissue repair and regeneration. Furthermore, our study corroborates others that also found that ALA accelerates the inflammatory phase in hyperglycemic rats, facilitating the early onset of the proliferation phase and increasing fibroblast proliferation, in addition to inducing angiogenesis [46].

The same trend was seen with collagen deposition, evaluated here by picrosirius staining (Figure 9a. We conducted this experiment only with the hyperglycemic group and observed that the ALA-treated group was further along in the healing process, showing mature fibers appearing as thicker, more organized bundles, while immature fibers appeared more diffuse and less aligned (Figure 9a,b). Furthermore, the ALA-treated group presents a greater amount of collagen type I fibers (red) compared to the vehicle-treated group (Figure 9e). Type I collagen plays a crucial role in wound healing, particularly during the final stages of tissue repair. It is the dominant collagen type in the skin and is synthesized by fibroblasts to replace the initial collagen type III. While collagen type III is initially deposited, type I collagen assumes the role of structural protein, contributing to the tensile strength of the scar tissue [47]. Thus, these data show that ALA plays an important role in restoring skin integrity.

## 5. Conclusions

As a whole, the results of this study indicate that the controlled delivery of ALA through a subcutaneously implanted osmotic pump improves the healing process in hyperglycemic mice by accelerating the inflammatory phase and promoting an earlier formation of granulation tissue facilitated by the balance between vimentin, CD31, and MMP-9 expression. Additionally, ALA modulates Bdnf, Ntrk2, Sox-10, Cntr, Cntr receptor, and Stat-3, genes associated with peripheral neurogenesis and gliogenesis. It may also enhance wound healing and nerve regeneration at the wound site in hyperglycemic mice. In non-hyperglycemic mice, ALA improves scar quality; however, it does not speed up wound healing, despite the positive modulation of some genes that are important in skin healing. The systemic delivery of ALA facilitated its controlled release over time; however, it did not change the overall lipid composition, suggesting that this method of administration is safe.

## 6. Study Limitations

Functional and nerve conduction studies need to clearly demonstrate the effects of ALA on nerve regeneration within the wound. Additionally, it would be valuable to investigate the spatial distribution of CD-31 and TGF-β1 proteins during the re-epithelialization process.

## Data Availability

The authors will provide the underlying data that supports the findings of this article upon request.

## Supplementary Materials

The following supporting information can be downloaded at: https://www.mdpi.com/article/10.3390/pharmaceutics17111427/s1, Supplementary Figure S1: Fasting blood glucose. Data expressed as mean ± SE (n = 4–5 per group). *p* < 0.05. Supplementary Figure S2: Heat map of lipid profile (medium- and long-chain saturated fatty acids) in scar tissue of non-hyperglycemic and hyperglycemic animals treated with ALA: Data are expressed as µg/mg tissue. N = 3–4 per group.

## Abbreviations

The following abbreviations are used in this manuscript:

ALA	Alpha-linolenic acid
BDNF	Brain-Derived Neurotrophic Factor
PECAM-1	Platelet endothelial cell adhesion molecule 1
MMP-9	Metalloproteinase
CNTF	Ciliary Neurotrophic Factor
CNTFR	Ciliary Neurotrophic Factor Receptor
IGF-1	Insulin-like Growth Factor
ITGA-5	Integrin Subunit Alpha 5
MUFA	Monounsaturated fatty acid
Ngfr	Nerve Growth Factor
NRK2	Neurotrophic Receptor Tyrosine Kinase 2
PUFA	Polyunsaturated Fatty Acid
SOX-10	SRY-Box Transcription Factor 10
STAT-3	Signal Transducer and Activator of Transcription 3
TGF-β1	Transforming Growth Factor Beta 1
VEGF	Vascular Endothelial Growth factor
FGF-1	Fibroblast Growth Factor
