# Supplementary material for: Therapeutic Effect of Alpha Linolenic Acid on Cutaneous Wound Healing in Hyperglycemic Mice: Involvement of Neurotrophins
Source: Pharmaceutics. 2025 Nov 4;17(11):1427. doi: 10.3390/pharmaceutics17111427 (PMC12655291; doi:10.3390/pharmaceutics17111427)
Supplement: Supplementary file 1 [file pharmaceutics-17-01427-s001.zip › pharmaceutics-3815629-supplementary.pdf]

Article

Therapeutic Effect of Alpha Linolenic Acid on Cutaneous Wound Healing in Hyperglycemic Mice: Involvement of Neurotrophins

Thais Paulino do Prado [1,2], Flávia Cristina Zanchetta [1,2], Aline Cristina Rosa Maria [3], Thaiane da Silva Rios [4], Guilherme Rossi de Assis-Mendonça [5], Maria Helena Melo Lima [1,2], Dennys Esper Correa Cintra [2,4], Joseane Morari [2], Lício A. Velloso [2,5] and Eliana P. Araújo [1,2,*]

Supplementary Materials

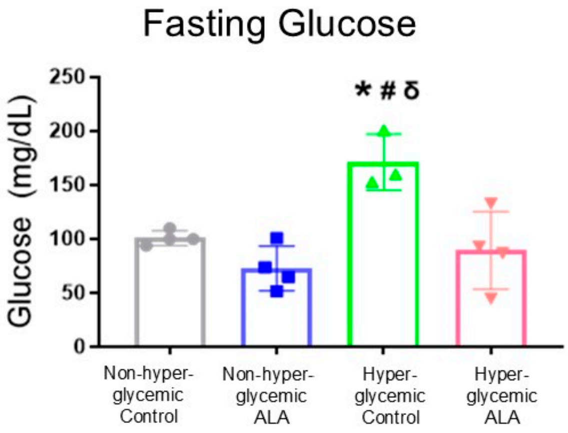

Supplementary Figure S1:. Fasting Blood Glucose: Data expressed as mean ± standard error, N = 4, $p < 0.05$; # Hyperglycemic Control vs. Non-Hyperglycemic Control; δ Hyperglycemic Control vs. Non-Hyperglycemic ALA; δ Hyperglycemic Control vs. Hyperglycemic ALA. This graph is representative of measurements performed every two days throughout the wound healing experiment.

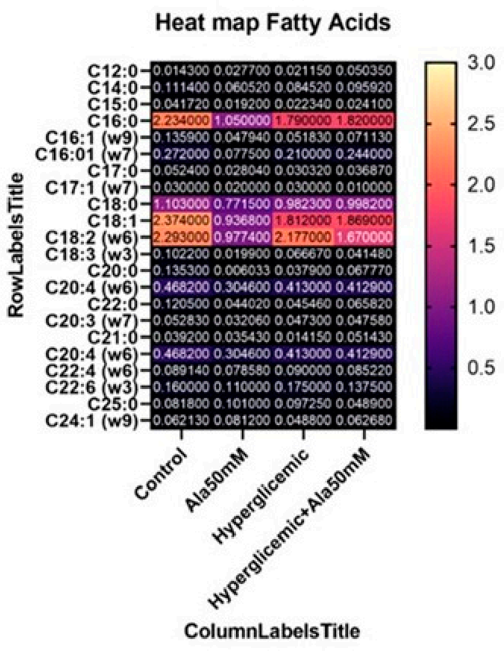

Supplementary Figure S2:. Heat map of lipid profile (medium- and long-chain saturated fatty acids) in scar tissue of non-hyperglycemic and hyperglycemic animals treated with ALA: Data are expressed as μg/mg tissue. N = 3–4 per group.
